# Evaluation of the registry DIALYREG for the assessment of continuous renal replacement techniques in the critically ill patient

**DOI:** 10.1038/s41598-023-32795-y

**Published:** 2023-04-20

**Authors:** M. González-Fernández, N. Quílez-Trasobares, J. A. Barea-Mendoza, Z. Molina-Collado, D. Arias-Verdú, J. Barrueco-Francioni, G. Seller-Pérez, M. E. Herrera-Gutiérrez, J. A. Sánchez-Izquierdo Riera

**Affiliations:** 1 Department of Intensive Care Medicine, University Hospital 12 de Octubre, Madrid, Spain; 2 Department of Intensive Care Medicine, Regional University Hospital of Malaga, Malaga, Spain; 3 Instituto de Investigación Biomédica de Málaga (IBIMA), Málaga, Spain; 4 Departamento de Medicina y Dermatología, University of Malaga, Malaga, Spain

**Keywords:** Continuous renal replacement therapy, Acute kidney injury, Epidemiology

## Abstract

Continuous renal replacement techniques (CRRT) can induce complications and monitoring is crucial to ensure patient safety. We designed a prospective multicenter observational and descriptive study using the DIALYREG registry, an online database located on a REDCap web-based platform that allows real-time data analysis. Our main objective was to identify CRRT-related complications in our intensive care units (ICUs) and implement security measures accordingly. From January 2019 to December 2020, we included 323 patients with admission diagnoses of medical illness (54%), sepsis (24%), postoperative care (20%), and trauma (2%). CRRT indications were homeostasis (42%), oliguria (26%), fluid overload (15%), and hemodynamic optimization (13%). The median initial therapy dose was 30 ml/kg/h (IQR 25–40), and dynamic adjustment was performed in 61% of the treatments. Sets were anticoagulated with heparin (40%), citrate (38%) or no anticoagulation (22%). Citrate anticoagulation had several advantages: more frequent dynamic CRRT dose adjustment (77% vs. 58% with heparin and 56% without anticoagulation, p < 0.05), longer duration of set (median of 55 h, IQR 24–72 vs. 23 h, IQR 12–48 with heparin and 12 h, IQR 12–31 without anticoagulation, p < 0.05), less clotting of the set (26% vs. 46.7% with heparin, p < 0.05), and lower incidence of hypophosphatemia (1% citrate vs. 6% with heparin and 5% without anticoagulation). It was also safe and effective in subgroup analysis of patients with liver disease or sepsis. The main global complications were hypothermia (16%), hypophosphatemia (13%) and metabolic acidosis (10%). Weaning of the therapy was achieved through early discontinuation (56%), nocturnal therapy transition (26%) and progressive SLED (18%). 52% of the patients were discharged from the hospital, while 43% died in the ICU and 5% died during hospitalization. We can conclude that the DIALYREG registry is a feasible tool for real-time control of CRRT in our ICU.

## Introduction

About half of critically ill patients will suffer acute kidney injury (AKI) and 20% will require continuous renal replacement therapy (CRRT) within the first week of admission in ICU^1,2^. This situation is associated with a mortality rate of 50%, and the accomplishment of best practices in the management of these techniques is essential to improve outcomes in a highly vulnerable population.

The increasing incidence of AKI treated with CRRT and the improvements in safety and depurative efficiency of the therapies has promoted an expansion of the indications and technical modalities of CRRT in Critical Care. It has also forced us to deepen our knowledge in this setting and to give specific training to all the professionals involved in its management.

Despite the high incidence of AKI in ICU, the frequent use of CRRT, and the complexity of the process, few specific registries evaluate the use of these techniques in critically ill adult patients^3^. For this reason we developed a multicenter registry in ICU that would allow us to analyze the demographic and epidemiological characteristics of patients receiving CRRT, to detect and correct problems in the application of these therapies, and to identify the advantages and disadvantages of different anticoagulation strategies.

## Methods

### Study design and setting

Our present study is a prospective, multicenter, observational study from the DIALYREG registry. This is an online database located on a REDCap web-based platform providing legal guarantees regarding security and confidentiality and allowing for real-time data analysis.

The study was conducted in the Intensive Care Services of two third-level hospitals in Spain: University Hospital 12 de Octubre and Regional University Hospital of Malaga. The University Hospital 12 de Octubre serves as a specialized regional referral center for the south region of Madrid. It provides care for a population of 500,000 people, with 1300 beds for acute hospitalization, 38 surgical wards, and 2 heliports; as well as a polyvalent ICU with 17 beds and 650 admissions of diverse complexity annually. The Regional University Hospital of Malaga also serves as the regional referral center for the Malaga province. It provides care for a population of 400,000 people with 1017 beds for acute hospitalization, including 60 ICU beds receiving 2200 admissions annually.

The inclusion criteria for the registry were: adult patients (age > 18 years old) admitted to the ICU with AKI who underwent CRRT. Patients that don’t meet these conditions were excluded. We collected data from January 2019 to December 2020. The variables analyzed are shown in Table 1. This study followed the Strengthening the Reporting of Observational Studies in Epidemiology (STROBE) reporting guideline.

**Table 1:** Collected variables from DIALYREG registry.

Demographic	
ICU admission	ICU admission criteria Date of ICU admission and discharge Hospital and ICU discharge situation
CRRT characteristics	Indication of therapy Duration of therapy Initial modality Initial and final dose Dynamic adjustment Phosphate fluids utilization Reason for removal Weaning method Associated complications
Hemofilter characteristics	Total number of hemofilters Filter duration Hours lost during change Type of membrane Type of anticoagulation Number of therapy changes Reason for removal
Catheter characteristics	Total number of catheters Diameter Length Duration Type of catheter Vascular access Complications Reason for removal

### CRRT protocol

CRRT was performed using Prismaflex or Prismax Hemofiltration (HF) Set Systems (Baxter Healthcare). The therapy was conducted and adjusted by intensivists according to the local protocol and the patient’s specific needs, with ICU nurses assembling the circuit. The predominant modality of use was continuous venovenous hemodiafiltration (CVVHDF) with a blood flow rate of 150–200 ml/min and an initial therapy dose of 35 ml/kg/h distributed at 50% between dialysis and hemofiltration. Replacement fluids were infused post-filter. We used fluids with low potassium and without phosphate in patients with severe hyperkalemia and fluids with 4 mmol/l potassium and 1.2 mmol/l phosphate in the other cases. Protocolized change of the hemofilter was performed when transmembrane pressure reached 200 mmHg or at 72 h of utilization (maximum filter lifespan according to manufacturer’s warranty). Our CRRT protocol practices a dynamic dose approach, adjusting the treatment to the patient’s situation^4^.

We used unfractionated heparin at 500 IU/h (with or without associated epoprostenol) for anticoagulation of CRRT in patients without significant thrombopenia (> 75,000 platelets per microliter of blood), coagulopathy or high bleeding risk. We used citrate if any of the previous situations existed or if clotting was frequent (> 2 set/24 h) with other anticoagulation methods. In some cases, it was possible not to use any anticoagulation method, taking into account the specific risks and benefits of the patient.

In citrate setting, we used a standard therapy with a blood flow rate of 140 ml/min, dialysis of 1500 ml/h, and replacement of 300 ml/h (calculated blood citrate concentration of 3 mmol/l). Monitoring of total calcium and ionized calcium is set one hour after the CRRT and every 8 h after that, to maintain ionized calcium level between 0.25 and 0.35 at the circuit and between 0.9 and 1.1 in patient’s blood.

The acid–base status, uremia, and electrolytes were also monitored daily and more frequent blood analyses were undertaken if any complications developed.

### Statistical analysis

The categorical variables are presented as incidence and percentage. The difference between categorical variables was assessed by using the chi-square test or Fischer’s exact probability test. The continuous variables with normal distribution and non-normal distribution were presented as mean ± SD (standard deviation) and median with IQR (interquartile range), respectively. Comparison between two groups was performed using the Student’s t-test for normal distribution continuous variables and Wilcoxon signed-rank test for non-normal distribution variables. p < 0.05 was considered as statistically significant. All the statistical analysis was performed by using SPSS software (IBM SPSS 22.0).

### Ethical considerations

DIALYREG registry collection data and analysis was conducted in accordance with the Helsinki Declaration and was approved by the Ethics Committee of University Hospital 12 de Octubre and the Ethics Committee of Regional University Hospital of Malaga. Informed consent was obtained from the patients or their family members during the admission to ICU.

## Results

### Baseline characteristics

Between January 2019 and December 2020, 323 patients were enrolled. The admission diagnosis included: medical illness (54%), sepsis (24%), postoperative care (20%), and trauma (2%). The main indication for CRRT was homeostasis (42%) followed by oliguria (26%), fluid overload (15%), and hemodynamic optimization (13%).

The median of catheters per patient was 1 (IQR 1–1) with a median duration of 5 days (IQR 3–9) and a total of 424 catheters were used. 82% of catheters were coaxial dual lumen and the preferential vascular access was right femoral vein (57%). Complications due to cannulation or the catheter itself were infection (1.7%), hematoma (1.4%), and thrombosis (1.2%).

The median number of hemofilters per patient was 3 (IQR 2–6) with a mean set duration of 31 h (SD 24) resulting in a total of 1316 hemofilters. The median initial therapy was 30 ml per kilogram per hour (IQR 25–40) with dynamic adjustment in 61% of the treatments.

### Anticoagulation (Table 2)

**Table 2:** Characteristics and complications of CRRT by anticoagulation type.

	Citrate	Heparin	Without anticoagulation	
Number of patients	123	129	71	
Total percentage	38%	40%	22%	
Initial dose	32 ml/kg/h	33 ml/kg/h	29 ml/kg/h	
Final dose	28 ml/kg/h	30 ml/kg/h	28 ml/kg/h	
Dynamic adjustment	76%	58%	54%	p < 0.05
Hemofilter duration	55 h (IQR 24–72)	23 h (IQR 12–48)	12 h (IQR 12–31)	p < 0.05
Lost due to clotting	7%	16%	12%	
Hypothermia	4%	5%	6%	
Hypokalemia	3%	1%	1%	
Hypomagnesemia	6.6%	2%	2%	p < 0.05
Hypophosphatemia	1%	5%	6%	p < 0.05
Metabolic acidosis	2.89%	3%	3.1%	

The anticoagulation used for the sets was: heparin (40%), citrate (38%), and no anticoagulation (22%). The initial and final dose of the therapy was similar between the three groups, but dynamic adjustment was more frequent in the citrate group (77%) than in the heparin (58%) and no-anticoagulation (56%) groups (p < 0.05). The median set duration was longer in the citrate group (55 h, IQR 24–72) than in the heparin (23 h, IQR 12–48) and no-anticoagulation one (12 h, IQR 12–31), (p < 0.05). Eliminating SLED (Sustained Low-Efficiency Dialysis), the median set duration was the same in the citrate group but longer in the heparin group (38 h, IQR 21–52) and also the no-anticoagulation group (26 h, IQR 13–50), (p < 0.05). Set clotting was less frequent in the citrate group (26%) than in the heparin group (44%) (p < 0.05).

The differences between therapy complications due to hypothermia, hypokalemia, metabolic acidosis or metabolic alkalosis were not statistically significant. We identified a higher incidence of hypophosphatemia in the heparin (5%) and without anticoagulation (6%) groups than in the citrate group (1%). Also a higher incidence of hypomagnesemia in the citrate group than in the other two groups (6.6% vs. 2% with p < 0.05).

### Outcomes

The main reason for discontinuation of the therapy was recovery of renal function (52%), followed by death of the patient (33%) and transition to intermittent renal replacement techniques (11%). Interruption of therapy was due to planned interruption (36%), set coagulation (35%) or ending of the treatment (22%). The main global complications were hypothermia (16%), hypophosphatemia (13%), metabolic acidosis (10%), hypocalcemia (6%) and hypokalemia (5%).

Regional University Hospital of Malaga performed an initial analysis after the first eight months of data collection and detected deviations from the hospital protocol that resulted in an increase in CRRT complications. They developed a bundle of corrective actions and reevaluated their practice after a further three months. They identified an increase in the use of citrate (from 16.7 to 34.4%, p < 0.05), a lower percentage of therapy’s hours lost (from 17.4 to 3.7%, p < 0.05), and lower incidence of hypercalcemia (15.2 vs. 6.3%, p < 0.05) and hypophosphatemia (27.5 vs. 17.7%, p < 0.05).

Weaning of the therapy was performed by discontinuation without diuretics in 43% of the cases, nocturnal therapy transition in 26%, progressive SLED in 18% and discontinuation with diuretics in 13% of cases.

52% of the patients were discharged from the hospital, while 43% died in the ICU and 5% died during hospitalization.

Efficiency evaluation according to standard commercialization fees of sets and fluids, therapy dose and set duration (eliminating SLED) led to a similar 72-h-therapy cost for a 70 kg patient (€827 with citrate vs. €783 with heparin) between different anticoagulation strategies in our series.

### Specific subgroups

#### Patients with liver disease

We analyzed data from patients with liver disease in our series, resulting in a total of 37 patients and 145 hemofilters (Table 3). They presented with liver cirrhosis in 54%, postoperative care for liver transplantation in 37%, and acute liver failure in 7% of cases. The median age was 55 years (IQR 46–66) and there was male predominance (78%). The indication for CRRT was homeostasis (52%), fluid overload (26%), oliguria (11%), and hemodynamic optimization (11%).

**Table 3:** General characteristics of CRRT by pathology subgroup in our series.

	Global	Liver disease	Sepsis
Number of patients	323	37	75
Number of hemofilters	1318	145	281
Pathology	Medical illness 54% Sepsis 24% Postoperative 20% Trauma 2%	Cirrhosis 54% Post-liver transplantation 37% Acute liver failure 7%	Respiratory 37% Abdominal 22% Urinary 15% Intravascular 11% Skin and soft tissue 9% CNS 3% Unknown 3%
Indication	Homeostasis 42% Oliguria 26% Fluid overload 15% Hemodynamics 13%	Homeostasis 52% Oliguria 11% Fluid overload 26% Hemodynamics 11%	Homeostasis 34% Oliguria 34% Fluid overload 19% Hemodynamics 23%
Anticoagulation	Heparin 40% Citrate 38% Without anticoagulation 22%	Heparin 39% Citrate 36% Without anticoagulation 25%	Heparin 27% Citrate 26% Without anticoagulation 47%

The anticoagulation of the sets was: 39% heparin, 36% citrate, and 25% without anticoagulation. Discontinuation of the therapy was due to set clotting in 17% of the no-anticoagulation hemofilters, 9% with heparin, and 3% with citrate. There were no statistically significant differences between therapy complications in the three groups of anticoagulation. Citrate’s posology showed important stability without adjustments in the calcium dose in 77% of the cases, with only one change in 2% and two changes in 16%. There were no cases of citrate accumulation, bleeding complications, or transfusion requirements related to the technique.

#### Patients with sepsis

We analyzed data from the patients with sepsis in our series (Table 3): 75 patients and 281 hemofilters. The median age was 59 years (IQR 48–68) with a male predominance (60%). The source of sepsis was: respiratory 37%, abdominal 22%, urinary 15%, intravascular 11%, skin and soft tissues 9%, central nervous system 3%, and unknown 3%. Mean SOFA score was 9.3 (SD 3.8), mean SAPS II was 59 (SD 16.25) and mean APACHE was 25 (SD 7.08). The indication of therapy was homeostasis (34%), oliguria (34%), hemodynamic optimization (23%), and fluid overload (9%). Hemodynamic optimization was a more frequent indication in patients with sepsis and fluid overload was a less frequent indication than in patients without sepsis (44% homeostasis, 28% oliguria, 9% hemodynamic optimization, and 19% fluid overload, p < 0.05). Initial dose therapy was 35 ml/kg/h (IQR 25–40) with dynamic adjustment in 60% of the subgroup. A conventional polyethylene membrane was used in 86% of the treatments, while high cut-off membrane was used in 11% and hemoadsorption with Oxiris® membrane in 3%.

The choice of anticoagulation of the sets was: 27% heparin, 26% citrate, and 47% without anticoagulation. This distribution led to a non-significant tendency towards not using anticoagulation in septic patients compared to non-septic patients, probably due to the higher use of high-flow CRRT in this clinical setting, without resulting in a higher proportion of set clotting. In the subgroup of patients with sepsis and citrate, there was no statistically significant differences in complications, however we did identify 2 cases of citrate accumulation.

Other complications of the therapy (hypothermia, ionic or acid–base disturbances, transfusion requirements) as well as reasons for removal and weaning methods in septic patients were all similar to patients without sepsis.

Survival of this subgroup of patients was 50% with a rate of mortality of 49% during ICU stay and 1% during hospitalization.

## Discussion

DIALYREG allowed us to understand our population from an epidemiological, clinical, and prognostic point of view. It provided evidence of educational, investigator, quality, and efficiency interest. With its use, we have been able to define the main indications, modalities, and doses applied to the CRRT in our ICUs and to resolve doubts about the use of these techniques in patients with specific associated risks, such as patients with liver disease or sepsis. We also detected hospital protocol deviations, as Regional Malaga Hospital’s analysis revealed. This strengthens the idea that data collection of CRRT applied to our patients builds a security climate that facilitates resource management^4,5^.

A better understanding of our practice has allowed us to endorse those aspects in which we adhere to clinical practice guidelines and international recommendations, such as the use of an average therapy dose of 30 ml/kg^6–10^, dynamic dosing^11^, or the low rate of therapy weaning with the use of diuretics (13%). It has also allowed us to identify areas for improvement, such as the predominance of right femoral access in our catheters despite right jugular access as preferable in terms of infection and thrombosis^12,13^. However, our infection and thrombosis rates were both less than 2%.

One of the objectives of our registry was to identify the advantages and disadvantages of the different anticoagulation methods that we apply to CRRT. Citrate-calcium based anticoagulation has several advantages over heparin, including regional application and its use in patients with thrombopenia and/or coagulopathy^14–16^. Its use is more complex than the other anticoagulation systems. Familiarity with the equipment is essential, with frequent monitoring of the ionic calcium both in the patient and in the circuit to ensure effective anticoagulation and prevent accumulation^17,18^. Thanks to DIALYREG, we identified a longer duration of citrate anticoagulated sets in our series and a smaller proportion of clotting hemofilters in this setting, these findings are consistent with other studies^19–21^. Associated complications were less frequent with citrate than with other anticoagulation methods, and we did not identify specific risks such as citrate accumulation or significant calcium alterations as described by Schneider et al.^18^. Its application in more demanding scenarios for CiCa depuration like patients with liver disease or sepsis also confirmed its security with thorough monitoring, as other authors subscribe^19–29^. The low proportion of acute liver failure in our series may have influenced these results, though^30^.

This establishes a thought about the percentage of citrate use seen in our series, with 36% in patients with liver disease or 26% in patients with sepsis are probably less than desired. 47% of no anticoagulation in patients with sepsis is probably excessive too. This probably relates to the use of high-flow therapies, in which citrate-calcium adjustment may be more difficult to do. Besides that, other studies establish a proportion of no anticoagulation between 30 and 60%^31–33^.

The duration of the sets shows a wide variability depending on the series. Starting from similar set durations under heparin in Hetzel et al.^34^ (26 h) or Oudemans-van Straaten et al.^35^ (27.5 h) compared to 23 h in our case, we have achieved longer durations with citrate than in previous studies: from 37.5 h in Hetzel et al.^34^ or 28.5 h in Oudemans-van Straaten et al.^35^ to 55 h in our case. Prolonged durations have also been described as Monchi et al.^36^ (81.4 h) and Kutsogiannis et al.^37^ (125.5 h) showed.

Efficiency evaluation of the techniques shows that they have a similar cost, so the more frequent clotting of filters may be the determining aspect. This results in a bigger healthcare workload and a potential harm to the patient, who misses effective therapy hours and may increase blood loss due to the non-returnable clotting of the set. It should also be considered that the ICU services involved in this study have extensive experience in CRRT therapies. Efficiency may be even more favorable to citrate in services with shorter patency of heparin anticoagulated circuits.

Due to the small sample size, we didn’t analyze specific therapies like hemoadsorption in patients with cytokine-release-syndrome after oncohematologic therapies, plasmapheresis in myasthenia gravis patients, or MARS as a bridge therapy to liver transplantation. The continuation of the registry and the inclusion of other hospitals may help us to acknowledge unusual pathologies with growing incidence and a greater risk of complications.

Our study has several limitations: First, the short period of inclusion (2 years, being one of them strongly affected by the COVID-19 pandemic). Second, although the evaluation of indications for CRRT was not an objective of our registry and the application of these techniques is performed according to our protocol, we did not collect AKI specific data before the initiation of the therapy to evaluate its appropriateness. Third, specific data related to the patient’s comorbidities or nephrotoxic exposure that may be of interest in prognosis on patient’s recuperation was not collected. Fourth, besides ICU and hospital mortality, functional data on quality of life, chronic kidney injury development, or hemodialysis dependency was not collected. Finally, it is a multicenter study but limited to just two hospitals, which also share a unified protocol of CRRT.

The benefits from the application of a multicenter registry in terms of security for our patients and the simplicity of the REDCap platform have driven us to present the registry at the Nephrology Group meeting of the Spanish Society of Intensive Medicine (SEMICYUC), in order to broaden its application to a National scope. We are also thinking about an international registry that could allow benchmarking strategies. The future of CRRT could integrate monitoring and laboratory data with therapy characteristics and might be able to detect and resolve complications from automated systems.

## Data Availability

The datasets used and analyzed during the current study are available from the corresponding author on reasonable request.

## Abbreviations

AKI: Acute kidney injury
CRRT: Continuous renal replacement therapy
CVVHDF: Continuous veno-venous hemodiafiltration
ICU: Intensive care unit
IQR: Interquartile range
MARS: Molecular adsorbent recirculating system
SAPS: Simplified acute physiology score
SEMICYUC: Spanish Society of Intensive Medicine and Coronary Care Units
SD: Standard deviation
SLED: Sustained low-efficiency dialysis
SOFA: Sequential organ failure assessment

## References

[1] Hoste E, Bagshaw S, Bellomo R, Cely CM, Colman R (2015). Epidemiology of acute kidney injury in critically ill patients: The multinational AKI-EPI study. Intens. Care Med..

[2] Herrera-Gutiérrez ME, Seller-pérez G, Sánchez-izquierdo-riera JA, Maynar-moliner J (2013). Prevalence of acute kidney injury in intensive care units: The “COrte de prevalencia de disFunción RenAl y DEpuración en críticos” point-prevalence multicenter study. J. Crit. Care.

[3] Sutherland SM, Goldstein SL, Alexander SR (2014). The prospective pediatric continuous renal replacement therapy (ppCRRT) registry: A critical appraisal. Pediatr. Nephrol..

[4] Maynar Moliner J, Honore PM, Sanchez-Izquierdo Riera JA, Herrera Gutierrezx ME, Spape HD (2012). Handling continuous renal replacement therapy-related adverse effects in intensive care unit patients: The dialytrauma concept. Blood Purif..

[5] Harris PA, Taylor R (2009). Research electronic data capture (REDCap) a metadata-driven methodology and workflow process for providing translational research informatics support. J. Biomed. Inf..

[6] Ronco C, Bellomo R, Homel P (2000). Effects of different doses in continuous veno-venous haemofiltration on outcomes of acute renal failure: A prospective randomised trial. Lancet.

[7] Bouman CS, Oudemans-Van Straaten HM (2002). Effects of early high volume continuous venovenous hemofiltration on survival and recovery of renal function in intensive care patients with acute renal failure: A prospective, randomized trial. Crit. Care Med..

[8] Tolwani AJ, Campbell RC, Stofan BS (2008). Standard versus highdose CVVHDF for ICU-related acute renal failure. J. Am. Soc. Nephrol..

[9] Palevsky PM, Zhang JH, Connor TZ, Chertow GM, Crowley ST, Choudhury D (2008). VA/NIH acute renal failure trial network: Intensity of renal support in critically ill patients with acute kidney injury. N. Engl. J. Med..

[10] Van Wert R, Friedrich JO, Scales DC (2010). High-dose renal replacement therapy for acute kidney injury: Systematic review and meta-analysis. Crit. Care Med..

[11] Kidney Disease Improving Global Outcome KDIGO (2012). Acute kidney injury work group: KDIGO clinical practice guideline for acute kidney injury. Kidney Int. Suppl..

[12] Oliver MJ, Callery SM, Thorpe KE, Schwab SJ, Churchill DN (2000). Risk of bacteremia from temporary hemodialysis catheters by site of insertion and duration of use: A prospective study. Kidney Int..

[13] Parienti JJ, Thirion M, Megarbane B (2008). Femoral vs jugular venous catheterization and risk of nosocomial events in adults requiring acute renal replacement therapy: A randomized controlled trial. JAMA.

[14] Kindgen-Milles D, Brandenburger T, Dimski T (2018). Regional citrate anticoagulation for continuous renal replacement therapy. Curr. Opin. Crit. Care.

[15] Betjes MG, van Oosterom D, van Agteren M, van de Wetering J (2007). Regional citrate versus heparin anticoagulation during venovenous hemofiltration in patients at low risk for bleeding: Similar hemofilter survival but significantly less bleeding. J. Nephrol..

[16] Li L (2021). Regional citrate anticoagulation vs no-anticoagulation for CRRT in hyperlactatemia patients with increased bleeding risk: A retrospective cohort study. Semin. Dial..

[17] Brophy PD, Somers MJ, Baum MA, Symons JM, McAfee N, Fortenberry JD (2005). Multicentre evaluation of anticoagulation in patients receiving continuous renal replacement therapy (CRRT). Nephrol. Dial. Transpl..

[18] Schneider AG, Journois D, Rimmelé T (2017). Complications of regional citrate anticoagulation: Accumulation or overload?. Crit. Care.

[19] O’Learly J, Greenberg C, Patton H, Caldwell S (2019). AGA clinical practice update: Coagulation in cirrhosis. Gastroenterology.

[20] Zhang W, Bai M, Yu Y, Li L, Zhao L, Sun S, Chen X (2019). Safety and efficacy of regional citrate anticoagulation for continuous renal replacement therapy in liver failure patients: A systematic review and meta-analysis. Crit. Care.

[21] Klingele M, Stadler T, Fliser D, Speer T, Groesdonk HV, Raddatz A (2017). Long-term continuous renal replacement therapy and anticoagulation with citrate in critically ill patients with severe liver dysfunction. Crit. Care.

[22] Schultheiss C, Saugel B, Phillip V, Thies P, Noe S, Mayr U (2012). Continuous venovenous hemodialysis with regional citrate anticoagulation in patients with liver failure: A prospective observational study. Crit. Care.

[23] Lahmer T, Messer M, Rasch S, Beitz A, Schnappauf C, Schmid RM (2015). Sustained low-efficiency dialysis with regional citrate anticoagulation in medical intensive care unit patients with liver failure: A prospective study. J. Crit. Care.

[24] Slowinski T, Morgera S, Joannidis M, Henneberg T, Stocker R, Helset E (2015). Safety and efficacy of regional citrate anticoagulation in continuous venovenous hemodialysis in the presence of liver failure: The liver citrate anticoagulation threshold (L-CAT) observational study. Crit. Care.

[25] Zhang (2019). Safety and efficacy of regional citrate anticoagulation for continuous renal replacement therapy in liver failure patients: A systematic review and meta-analysis. Crit. Care.

[26] European Association for the Study of the Liver (2018). EASL Clinical Practice Guidelines for the management of patients with decompensated cirrhosis. J. Hepatol..

[27] Younossi Z, Anstee QM, Marietti M, Hardy T, Henry L, Eslam M (2018). Global burden of NAFLD and NASH: Trends, predictions, risk factors and prevention. Nat. Rev. Gastroenterol. Hepatol..

[28] MacDonald A, Nadim M, Durand F, Karvellas C (2019). Acute kidney injury in cirrhosis: Implications for liver transplantation. Curr. Opin. Crit. Care.

[29] Davenport A, Sheikh M, Lamb E, Agarwal B, Jalan R (2017). Acute kidney injury in acute-on-chronic liver failure: Where does hepatorenal syndrome fit?. Kidney Int..

[30] Thanapongsatorn P (2022). Citrate pharmacokinetics in critically ill liver failure patients receiving CRRT. Sci. Rep..

[31] Tan HK, Baldwin I, Bellomo R (2000). Continuous veno-venous hemofiltration without anticoagulation in high-risk patients. Intens. Care Med..

[32] Bai M, Zhou M, He L (2015). Citrate versus heparin anticoagulation for continuous renal replacement therapy: An updated meta-analysis of RCTs. ICM.

[33] Liu C, Mao Z, Kang H (2016). Regional citrate versus heparin anticoagulation for continuous renal replacement therapy in critically ill patients: A meta-analysis with trial sequential analysis of randomized controlled trials. Crit. Care.

[34] Hetzel GR, Schmitz M, Wissing H, Ries W, Schott G, Heering PJ (2011). Regional citrate versus systemic heparin for anticoagulation in critically ill patients on continuous venovenous haemofiltration: A prospective randomized multicentre trial. Nephrol. Dial. Transplant.

[35] Oudemans-van Straaten HM, Bosman RJ, Koopmans M, van der Voort PH, Wester JP, van der Spoel JI (2009). Citrate anticoagulation for continuous venovenous hemofiltration. Crit. Care Med..

[36] Monchi M, Berghmans D, Ledoux D, Canivet JL, Dubois B, Damas P (2004). Citrate vs heparin for anticoagulation in continuous venovenous hemofiltration: A prospective randomized study. Intens. Care Med..

[37] Kutsogiannis DJ, Gibney RT, Stollery D, Gao J (2005). Regional citrate versus systemic heparin anticoagulation for continuous renal replacement in critically ill patients. Kidney Int..

